# A Baltimore Letter

**Published:** 1880-09

**Authors:** 


A BALTIMORE LETTER.

"O wad some pow'r the giftie gie us
*To see oursels as others see us!*"

The following letter is copied from the *Cincinnati Lancet and Clinic*, of August 14th, 1880, for the benefit of all concerned, and to correct a few errors into which the writer was led in regard to medical schools here. But to the letter first:

THE CITY OF BALTIMORE.

BALTIMORE, August 6, 1880.

*Editors Lancet and Clinic:*

The thrift and prosperity which has attended this city of monuments of late, and continues to extend its glow over her future, has not favorably affected the medical profession within her limits. On the contrary, if one were to judge of the health of her mercantile and manufacturing interest by the state of her professional interest the former would be set down as decidedly bad. Your city, the cradle of diploma mills, and the home of eclectics, homœopaths, etc., can boast, however, of three regular medical colleges conducted on wholesome principles; but this great city, claiming a hundred thousand more in population, cannot be proud of a single institution where medicine is taught in a thorough and dignified manner. Why are things professional so degraded—why has the physician so compromised his dignity that even laymen cease to respect him? Competition has often been called the life of trade, but without honor it is certainly the death of it. Up to the close of the war medical education here was good; since that time it has been indifferent, and may now be called bad. The starting point of the trouble must have had its origin in the advent of Edward Warren to the Medical Department of the University of Maryland during the session of 1860-61. This gentleman, being from the South, (Edenton, N. C.,) was sought after because of his attraction for Southern students, and he was elected to the chair of materia medica and therapeutics the same year that Hammond of New York was chosen to the chair of anatomy. Warren delivered but one course of lectures—which, by the way, were criticised at the time as being almost a repetition of the works of Martin Payne—and afterwards abandoned the college and went South. Notwithstanding the fact that he was repeatedly urged to return and take his place at the school, he remained South until the trustees, believing he would not return, filled the chair by electing Dr. Chew. When the last named gentleman had completed his first course of lectures, Dr. Warren returned and demanded his chair. The chair was, however, not forthcoming and the Doctor immediately resolved on taken vengeance. The defunct charter of the old Washington College was brought forth, and from this relic rose a structure in 1866 which fixed itself like a thorn in the side of the old institution. The South was appealed to and response came which filled the halls of the new institution and brought regrets to the old.

But this prosperity and harmony was not of long duration ; for internal strife soon commenced and Warren appeared as champion of another new departure. In 1868 the College of Physicians and Surgeons of Baltimore came to life with Warren in the role of accoucheur, nurse, and protector. He remained in this school, conducting it pretty much as he liked, until he left the country. Up to a short time ago these three institutions of learning drew their slow lengths annually along, each knifing its neighbor whenever an opportunity offered ; but now the field is left to two of them, the Washington College having fallen a victim to the more prosperous College of Physicians and Surgeons. Notwithstanding the fact that the Beneficiary System was a prominent feature in the working of this institution, it pined away and finally succumbed to the lack of patronage. "What is this beneficiary. system?" I asked. It is an arrangement by which so many students are admitted from each congressional district free of charge, and others, clubbing together, are allowed to come in at reduced rates. Say, for instance, the aggregate of the fees being fifty dollars (as would appear in the announcement,) a club of six would be received for a hundred and fifty dollars. This clubbing system, from what I can gather, was not confined to any one of the colleges but used by them all, and, in fact, it has not yet been discarded.

*No one pretends to say for a moment* that the Medical Department of the University *does not stand much higher* than the other school, but it is said that some of the faculty of the first named institution do not adhere strictly to the code of ethics, as regards petty advertising. An ex-Dean is said to indulge in this phase of innocence (?) without even exciting the suspicion much less the indignation of his comrades.

The classes at the College of Physicians and Surgeons are about twice as large as those at the University, but this is no word in their favor unless they are thorough in teaching and careful in their final examinations. It would be unnatural to expect such a happy state to exist when other conditions are so imperfect. Indeed such has been the character of the professors and graduates of the Baltimore schools that the Homœopaths found it necessary to introduce into the Legislature last year "a bill to provide against quackery in the State of Maryland;" and it was only the most earnest efforts of the University people that prevented it from becoming a law. This of itself is quite enough to indicate the odium attached to medical teaching in this locality.

The buildings of the Johns Hopkins' University are at present undergoing erection, and it is understood that 2 years more will elapse before their completion. The chairs will then be filled in the same gradual manner and Baltimore may begin to hope for better things. Every ambitious eye in the city is turned in that direction, but they will look in vain; for the probability is that most of the timber will be brought from other places.

The medical societies of Baltimore are three in number, The Clinical Society, East Baltimore Medical Society, and the Baltimore Academy of Medicine. The latter admits no one to membership who has not been in practice ten years. In addition to this feature a prize of a hundred dollars is given annually for the encouragement of original research. The last prize was taken by Dr. Councilman of this city, but I have not been able to learn the subject of his essay.

It is here as in Cincinnati, there are no such things as specialties. Every surgeon treats hay-fever or the measles, and the oculist does not hesitate to attend a case of colic. The chief complaint is that there are so many cheap doctors. Some men long in practice and wealthy seem to have a mania for popularity, and in the extremes they go to multiply their patients they often rob the younger practitioner of the means of subsistence. What young physician can afford to practice purely for the love of his profession, and how can he survive when the old physicians around him give away their services? This would not be objected to if it brought no one inconvenience and was really a measure for good, but the motives seem to be purely selfish and such as could only happen in a community where the profession were in a demoralized condition. But there are always individuals whose good stands out and enables us to judge of the ill of others. Baltimore is not lacking in such men no matter how much it may abound in less worthy ones. R. B. D.

Comment. Though hostile to the University of Maryland, Dr. Warren *was not* the originator of the movement that resulted in the revival of the Washington University, nor of that which gave birth to the College of Physicians and Surgeons. He became connected with both, soon after the idea of their establishment began to assume practical shape; and that *he ruled* either, is a great mistake. That *he* desired to do so, there is probably no doubt. But no one familiar with the facts, who knows him, ever arrogated to him, the origination of those schools, unless he did so himself, or it was claimed for him by one of the few satelites that regarded him as their "bright particular star!" The originator of those schools never designed nor intended them as *enemies* of the University of Maryland. The mistake in the date of origin of the College of Physicians and Surgeons, is probably typographical, and is unimportant. The size of the classes in that school is not wholly due to the "beneficiary," and "clubbing systems" prolific as they undoubtedly are in developing numbers; but to a more reprehensible one even, if the testimony of living witnesses can be relied upon, viz: that of *inviting* persons through printed circulars, or blanks, to be *filled* by themselves or friends, to become matriculants in the school, at almost nominal rates or charges!

Without a really eminent man in its faculty, or one that has been a "medical professor" a decade of years, strange enough the roster calls for about one hundred students from the State of Pennsylvania alone!!

and that too while the time honored *Medical Department of the University of Pennsylvania*; and (the school created by the great McClellan,) the *Jefferson Medical College*, of Philadelphia; with their *world* renowned Professors, and *unsurpassed* facilities for medical instruction, still exist! And yet, while those schools generously extend a hearty greeting to all worthy and diligent seekers after knowledge! About a hundred names appeared from Pennsylvania, in the last catalogue of the College of *Physicians and Surgeons!* "Why is this, thus?" Further comment from us is unnecessary.

